# Comparison of working length control consistency between hand K-files and Mtwo NiTi rotary system

**DOI:** 10.4317/jced.52561

**Published:** 2016-04-01

**Authors:** Károly Krajczár, Enikő Varga, Gyula Marada, Sára Jeges, Vilmos Tóth

**Affiliations:** 1 DMD. Clinical Consultant. Dental School, Faculty of Medicine, University of Pécs, Hungary; 2 DMD. Private practice, Budapest, Hungary; 3 DMD. Assistant Professor. Dental School, Faculty of Medicine, University of Pécs, Hungary; 4 BSc, MSc, PhD. Professor. Department of Biostatistics and Health Informatics, Faculty of Health Sciences, University of Pécs, Hungary; 5 DMD, PhD. Professor. Dental School, Faculty of Medicine, University of Pécs, Hungary

## Abstract

**Background:**

The purpose of this study was to investigate the consistency of working length control between hand instrumentation in comparison to engine driven Mtwo nickel-titanium rotary files.

**Material and Methods:**

Forty extracted maxillary molars were selected and divided onto two parallel groups. The working lengths of the mesiobuccal root canals were estimated. The teeth were fixed in a phantom head. The root canal preparation was carried out group 1 (n=20) with hand K-files, (VDW, Munich, Germany) and group 2 (n=20) with Mtwo instruments (VDW, Munich, Germany). Vestibulo-oral and mesio-distal directional x-ray images were taken before the preparation with #10 K-file, inserted into the mesiobuccal root canal to the working length, and after preparation with #25, #30 and #40 files. Working lenght changes were detected with measurements between the radiological apex and the instrument tips.

**Results:**

In the Mtwo group a difference in the working competency (*p*<0.05) could be noticed only in the vestibulo-oral direction from #10 to #40 file. The hand instrument group showed a significant difference in working length competency for each larger file size (*p*<0.05) (ANOVA). Regression analysis in the hand instrumentation group indicated a working length decrease with a mean of 0,2 mm after each consecutive file size (*p*<0.01).

**Conclusions:**

The outcome of our trial indicated a high consistency in working length control for root canal preparation under simulated clinical condition using Mtwo rotary files. Mtwo NiTi rotary file did therefore proved to be more accurate in comparison to the conventional hand instrumentation.

** Key words:**Working length, Mtwo, nickel-titanium, hand preparation, engine driven preparation.

## Introduction

The biomechanical objectives of root canal preparation are to eliminate pulp tissue and infection from the root canal system and to develop a continuously tapering conical form while the apical foramen is maintained in its original shape and position (1). An accurate determination of the working length has a fundamental role in the long term success of endodontic treatment which has been highlighted in various literatures (2). Recent research has proven that it is more important what is removed from the root canal during cleaning, shaping and disinfection than what is placed in to the canals during obturation. An incorrect determination of the working length or the loss of it could result in either under-instrumentation or over-instrumentation of root canals. Under-instrumentation of the canals means that the tip of the file does not reach to the working length resulting in remnants of the pulp tissue to be left near the apex of the roots. Microbiological literature suggests that microorganisms migrate towards the source of nutrients, which in the case of infected necrotic pulps and asymptomatic apical periodontitis, are located at the apical third of the tooth. Therefore under-preparation of these canals would result in a less elimination of necrotic pulp and the microorganisms. The presence of the micro-organisms results in a higher likelihood of re-infection of the canals, leading to a significant decrease in the long term success rate compared to cases in which accurate working length was maintained. On the other hand, over- instrumentation leads to apical constriction enlargement with consecutive trauma to the periapical tissues, extrusion of infected material and destruction of the apical binding point for the root filling which again results in a lower success rate of the endodontic treatment (2-4).

In a clinical practice, the determination of working length and its control remains still a challenge. Root canal instrumentation can be clinically demanding as a result of indirect vision of the operating field, reduced tactile sense, and limited space for the instrument movement. In addition, any change in the working length during the root canal instrumentation is also an important measure to quantify the performance of an instrument (3,5). The advent of nickel-titanium (NiTi) rotary instrumentation has revolutionized root canal treatment by reducing operator fatigue, time required to finish the preparation and minimized procedural errors associated with root canal instrumentation (3,6,7). The use of NiTi rotary system has enabled clinicians to achieve a more predictable and efficient canal preparations (3).

The principal purpose of this trial was to explore and evaluate the consistency of working length control by a comparison between hand instrumentation with K-files and with Mtwo engine driven NiTi rotary files.

## Material and Methods

This was a controlled clinical trial involving two parallel groups of extracted teeth. Forty maxillary first and second molars, extracted due to periodontal reasons were selected for the experiment. Immediately after extraction all teeth were kept in 10% buffered formalin for at least 48 hours. Tissue fragments and calcified debris were removed from the teeth using scalers. The teeth were then given a number and rinsed in tap water. Pulp chambers were opened using a water-cooled, cylindrical diamond bur (Brasseler, Lemgo, Germany) in a handpiece. The root canals were copiously irrigated with 5 ml 2,5% sodium hypochlorite using a 2 ml sterile syringe and a 30-gauge needle.

The access cavity preparation and the root canal treatment were carried out by a general practitioner experienced in endodontic treatment and procedures. Only the mesiobuccal (MB) canals were instrumented in both groups; the second MB canals were not included in our study. Apical patency was maintained by inserting a #10 K-file (VDW, Munich, Germany) until the tip of the file reached the level of the major foramen. Proper positioning was verified with a stereomicroscope (Opmi Pico, Carl Zeiss, Oberkochen, Germany) at a magnification of 13,6x. The silicon stopper was gently adjusted to correspond to the adjacent cusp tip (coronal reference point). The length between the stopper and the tip of the file was measured using a digital caliper and recorded to be near 0.1 mm. The determination of working length (WL) was obtained by taking the measured length and subtracting 1 mm from it.

The teeth in both groups were then individually embedded into a cubical resin block, 30 mm in height and 20-20 mm in width (Vertex Self Curing, Vertex - Dental, Zeist, The Netherlands) using a template. To avoid resin penetration into the apical foramen, the apices of the MB roots were isolated with a small putty impression material pellet (diameter: 2-3mm).

A #10 K-file (8) was inserted into the MB root canal until the WL and preoperative radiographs were taken in both Buccal-Palatal (BP) (clinical) and Mesio-Distal (MD) (proximal) directions (Kodak RVG 6100, Kodak, Marne la Valle, France; Trophy Elitys, No. REX1009, Trophy, Marne la Valle, France, exposed with 60kV, 4 mA, 0.065 s). Resin blocks made a standardized position possible for radiographs in the BP and MD directions. The distance between the tip of the instrument and the radiographic apex was measured with Kodak Dental Imaging Software 6.8.6.0. (Kodak, Marne la Valle, France). Teeth were then randomly selected and divided in two parallel groups (n=20). The mean curvature of the two groups was similar (Hand instrumentation (group 1): BP direction: 21,6 ± 7,595 median= 23,5; MD direction: 12,7 ± 8,27 median=11,5. Mtwo instrumentation (group 2): BP direction: 20,6 ± 6,805 median=18,5; MD direction: 11,4 ± 6,385 median=10,5).

To simulate clinical conditions during root canal instrumentation, teeth were integrated into a maxillary arch model at the corresponding anatomical position together with an antagonist mandibular arch model in a dummy head fixed to a phantom torso (Frasaco Phantom Head 6/3, Frasaco, Tettnang, Germany). In the upper plastic jaw, the resin blocks were secured with the help of a screw.

The same practitioner prepared each tooth. After each instrumentation the root canals were irrigated with 5 ml 2,5% sodium hypochlorite solution in both groups. The WL was transferred on K-files, and Mtwo files with a digital caliper and marked with silicone stoppers.

In group 1, canal preparation was carried out by hand instrumentation. An ISO #10 K file was placed to the working length and a reciprocal reaming action was used until it fit loosely in the canal. Successively larger files were used until the apical portion of the canal was instrumented to ISO #40 K file. In group 2 the root canals were prepared with 10/.04, 15/.05, 20/.06, 25/.06, 30/.05, 35/.04 and 40/.04 Mtwo files (VDW, Munich, Germany) consecutively. Root canal preparation was carried out in strict accordance with manufacturer’s recommendations. An electric motor with torque control (VDW Silver, VDW, Munich,Germany) was utilized with the NiTi system. All rotary instruments were discarded after the first preparation.

Post instrumentation radiographs were taken after completion of preparation with the file sizes #25, #30, and #40 in order to determine if the working length had been maintained. This procedure was performed for both the K- and Mtwo files respectively. The operator was not informed about the radiological findings or the results of the measurement obtained in order to avoid any modifications to be made and continued to prepare the canals according to the working length determined initially.

During the radiological assessment for each file the distance between the tip of the instrument and the radiographic apex was measured, and the differences between the preoperative and post instrumentation file tip – radiological apex distances was noted. These findings were analyzed statistically by analysis of variance (ANOVA) at 0.05 significance and regression analysis. SPSS 17.0 software was used for calculations.

The study protocol was approved by the local research ethics committee.

## Results

Forty root canals were instrumented, measured and subjected to statistical analysis. The files were examined after the instrumentation of the root canals and no fracture or permanent deformation could be observed. Means and standard deviations of change of WL between groups are shown in  Table 1. Statistical analysis of the data by ANOVA showed a difference (*p*<0.05) between the two groups at each preparation file sizes. In group 2, rotary Mtwo preparation showed noticeable differences (*p*<0.05) on the BP radiographs just between #10 and #40 instrument sizes. The hand instrument group (group 1) showed a significant difference for all three preparation file sizes in radiographs of both directions (BP and MD).

**Table 1:**
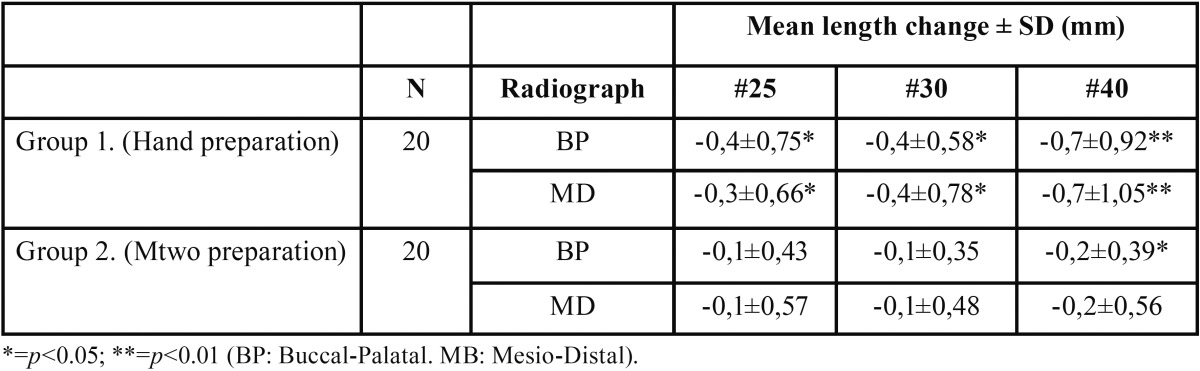
Comparison of working length changes measured on radiographs between hand instrumentation and Mtwo files by different preparation sizes.

The Regression analysis in the hand instrumentation group indicated a tendency toward a shortening of the real Working length with a mean of 0,2 mm by the insertion of each files. This means that each larger file size was inserted by a mean of 0,2 mm less into the canal suggesting an under preparation from the radiological apex (*p*<0.01) ( Table 1).

## Discussion

To date, no direct comparison of working length control has been reported between hand instrumentation and Mtwo rotary files under simulated clinical conditions. In our research we compared the working length control of preparations carried out by hand and Mtwo instruments under simulated clinical conditions on extracted teeth placed in phantom torso/head.

The literature suggest several limiting factors resulting in preparation errors, such as complex canal anatomy (9), lack of straight-line access (10), instrument design (5,11), instrumentation sequence (12), operator experience (13,14), rotational speed (14), and inadequate use of an irrigant or lubricant (3).

A potential problem with this study is the variation available on the market in blade design, cross section, tapering and manufacturing of instruments used for preparation of root canal (hand K-file versus rotary NiTi). This high variability makes the direct comparison of instruments ponderous and creates ground for pre-clinical comparative studies on their competency. Hand instruments have several disadvantages including limited flexibility and increased rigidity of mainly large stainless steel hand instruments. These properties of these files are supposed to be the main cause of procedural accidents (15). A better efficacy of rotary instruments in comparison with hand instruments has been shown (3,16-18). But there are some reports on the eligibility of Mtwo system (19-23).

The primary outcome of our trial was a significant difference in working length consistency between the hand files in comparison to a driven rotary instrumentation (Mtwo) technique in maintaining the determined working length.

In case of hand instrumentation the apical extension of the preparation was reduced simultaneously with increasing instrument sizes. Significant differences at Mtwo preparation could be noticed in the BP radiological direction between the #10 and #40 instrument sizes. However several studies have reported minor working length changes irrespectively of the type of instruments- hand, rotary NiTi- or techniques utilized (12,21,22,24-28). However, differences in the working length control using different preparation methods have been mentioned (29,30).

This trial was undertaken with appropriate positioned teeth in an upright positioned phantom torso, simulating the operating circumstances of an upper molar root canal treatment, considering and partly recreating the modifying factors arising from this position. Our findings in this trial indicate that Mtwo preparation enabled more accurate control and competency of the working length determined in comparison to hand files. A possible limitation of this study is the experience of the operator. Although in our study both the hand instrumentation and Mtwo preparations were carried out by the same general practitioner specialized in endodontics, is experience of operator a factor that cannot be controlled. Furthermore, within the dental care practice we must consider other uncontrollable factors, such as the patient’s movements, mouth opening ability. Consequently, further studies are needed to determine the most relevant modifying factors that could affect the quality of root canal preparation. However a close examination of the data we obtained suggests that the differences in maintaining the working length between the two methods are such that even a trial with a considerably larger sample and operators with different experiences would be unlikely to conclude any different result than our trial. We are therefore reasonably confident that the competency of rotary instrument (Mtwo) is more precise and generalized for endodontic treatment; however this will need confirmation with further clinical trials.

## References

[1] Schilder H (1974). Cleaning and shaping the root canal. Dent Clin North Am.

[2] Sjögren U, Hagglund B, Sundqvist G, Wing K (1990). Factors affecting the long-term results of endodontic treatment. J Endod.

[3] Peters OA (2004). Current challenges and concepts in the preparation of root canal systems: a review. J Endod.

[4] Schaeffer MA, White RR, Walton RE (2005). Determining the optimal obturation length: a meta-analysis of literature. J Endod.

[5] Iqbal MK, Banfield B, Lavorini A, Bachstein B (2007). A comparison of LightSpeed LS1 and LightSpeed LSX NiTi rotary instruments in apical transportation and length control in simulated root canals. J Endod.

[6] Ferraz CC, Gomes NV, Gomes BP, Zaia AA, Teixeira FB, Souza-Filho FJ (2001). Apical extrusion of debris and irrigants using two hand and three engine-driven instrumentation techniques. Int Endod J.

[7] Park H (2001). A comparison of Greater Taper files, ProFiles, and stainless steel files to shape curved root canals. Oral Surg Oral Med Oral Pathol Oral Radiol Endod.

[8] Piepenbring ME, Potter BJ, Weller RN, Loushine RJ (2000). Measurement of endodontic file lengths: a density profile plot analysis. J Endod.

[9] Haïkel Y, Serfaty R, Bateman G, Senger B, Allemann C (1999). Dynamic and cyclic fatigue of engine-driven rotary nickel-titanium endodontic instruments. J Endod.

[10] Bergmans L, Van Cleynenbreugel J, Beullens M, Wevers M, Van Meerbeek B, Lambrechts P (2003). Progressive versus constant tapered shaft design using NiTi rotary instruments. Int Endod J.

[11] Bishop K, Dummer PM (1997). A comparison of stainless steel Flexofiles and nickel-titanium NITiFlex files during the shaping of simulated canals. Int Endod J.

[12] Iqbal MK, Maggiore F, Suh B, Edwards KR, Kang J, Kim S (2003). Comparison of apical transportation in four Ni-Ti rotary instrumentation techniques. J Endod.

[13] Mandel E, Adib-Yazdi M, Benhamou LM, Lachkar T, Mesgouez C, Sobel M (1999). Rotary Ni-Ti profile systems for preparing curved canals in resin blocks: influence of operator on instrument breakage. Int Endod J.

[14] Yared GM, Bou Dagher FE, Machtou P (2001). Influence of rotational speed, torque and operator's proficiency on ProFile failures. Int Endod J.

[15] Torabinejad M (1994). Passive step-back technique. A sequential use of ultrasonic and hand instruments. Oral Surg Oral Med Oral Pathol.

[16] Glossen CR, Haller RH, Dove SB, del Rio CE (1995). A comparison of root canal preparations using Ni-Ti hand, Ni-Ti engine-driven, and K-Flex endodontic instruments. J Endod.

[17] Tan BT, Messer HH (2002). The quality of apical canal preparation using hand and rotary instruments with specific criteria for enlargement based on initial apical file size. J Endod.

[18] Liu SB, Fan B, Cheung GS, Peng B, Fan MW, Gutmann JL (2006). Cleaning effectiveness and shaping ability of rotary ProTaper compared with rotary GT and manual K-Flexofile. Am J Dent.

[19] Foschi F, Nucci C, Montebugnoli L, Marchionni S, Breschi L, Malagnino VA (2004). SEM evaluation of canal wall dentine following use of Mtwo and ProTaper NiTi rotary instruments. Int Endod J.

[20] Veltri M, Mollo A, Mantovani L, Pini P, Balleri P, Grandini S (2005). A comparative study of Endoflare–Hero Shaper and Mtwo NiTi instruments in the preparation of curved root canals. Int Endod J.

[21] Schäfer E, Erler M, Dammaschke T (2006). Comparative study on the shaping ability and cleaning efficiency of rotary Mtwo instruments. Part 1. Shaping ability in simulated curved canals. Int Endod J.

[22] Schäfer E, Erler M, Dammaschke T (2006). Comparative study on the shaping ability and cleaning efficiency of rotary Mtwo instruments. Part 2. Cleaning effectiveness and shaping ability in severely curved root canals of extracted teeth. Int Endod J.

[23] Martín-Micó M, Forner-Navarro L, Almenar-García A (2009). Modification of the working length after rotary instrumentation: a comparative study of four systems. Med Oral Patol Oral Cir Bucal.

[24] Thompson SA, Dummer PM (2000). Shaping ability of Hero 642 rotary nickel-titanium instruments in simulated root canals: Part 1. Int Endod J.

[25] Schäfer E, Lohmann D (2002). Efficiency of rotary nickel-titanium FlexMaster instruments compared with stainless steel hand K-Flexofile -- Part 1. Shaping ability in simulated curved canals. Int Endod J.

[26] Guelzow A, Stamm O, Martus P, Kielbassa AM (2005). Comparative study of six rotary nickel-titanium systems and hand instrumentation for root canal preparation. Int Endod J.

[27] Matwychuk MJ, Bowles WR, McClanahan SB, Hodges JS, Pesun IJ (2007). Shaping abilities of two different engine-driven rotary nickel titanium systems or stainless steel balanced-force technique in mandibular molars. J Endod.

[28] Karabucak B, Gatan AJ, Hsiao C, Iqbal MK (2010). A comparison of apical transportation and length control between EndoSequence and Guidance rotary instruments. J Endod.

[29] Schäfer E, Vlassis M (2004). Comparative investigation of two rotary nickel–titanium instruments: ProTaper versus RaCe. Part 1. Shaping ability in simulated curved canals. Int Endod J.

[30] Iqbal MK, Floratos S, Hsu YK, Karabucak B (2010). An in vitro comparison of Profile GT and GTX nickel-titanium rotary instruments in apical transportation and length control in mandibular molar. J Endod.

